# Human CLA^+^ Memory T Cell and Cytokines in Psoriasis

**DOI:** 10.3389/fmed.2021.731911

**Published:** 2021-10-29

**Authors:** Carmen de Jesús-Gil, Lídia Sans-de San Nicolàs, Irene García-Jiménez, Marta Ferran, Ramon M. Pujol, Luis F. Santamaria-Babí

**Affiliations:** ^1^Translational Immunology, Department of Cellular Biology, Physiology and Immunology, Faculty of Biology, Universitat de Barcelona, Parc Científic de Barcelona, Barcelona, Spain; ^2^Department of Dermatology, Hospital del Mar (Institut Hospital del Mar d'Investigacions Mèdiques), Universitat Autònoma de Barcelona, Barcelona, Spain

**Keywords:** psoriasis, CLA^+^ T cell, IL17A, interleukins, IL17F, IL-9, IL-15

## Abstract

Psoriasis is a common inflammatory skin condition resulting from the interplay between epidermal keratinocytes and immunological cellular components. This sustained inflammation is essentially driven by pro-inflammatory cytokines with the IL-23/IL-17 axis playing a critical central role, as proved by the clinical efficacy of their blockade in patients. Among all the CD45R0^+^ memory T cell subsets, those with special tropism for cutaneous tissues are identified by the expression of the Cutaneous Lymphocyte-associated Antigen (CLA) carbohydrate on their surface, that is induced during T cell maturation particularly in the skin-draining lymph nodes. Because of their ability to recirculate between the skin and blood, circulating CLA^+^ memory T cells reflect the immune abnormalities found in different human cutaneous conditions, such as psoriasis. Based on this premise, studying the effect of different environmental microbial triggers and psoriatic lesional cytokines on CLA^+^ memory T cells, in the presence of autologous epidermal cells from patients, revealed important IL-17 cytokines responses that are likely to enhance the pro-inflammatory loop underlying the development of psoriatic lesions. The goal of this mini-review is to present latest data regarding cytokines implicated in plaque and guttate psoriasis immunopathogenesis from the prism of CLA^+^ memory T cells, that are specifically related to the cutaneous immune system.

## Introduction

The regulation of cytokine production and signaling in chronic cutaneous inflammation is influenced by genetic and environmental factors. This minireview focuses on human psoriasis, both plaque and guttate forms of the disease, and the cytokine production by human circulating CLA^+^ memory T cells, a relevant subset of memory T cells associated to the regional cutaneous immune system. In translational research in dermatology, there is a need to develop new approaches beyond animal models and *in vitro* studies (1, 2), since the results in those models are not always translated into the clinic (3). For instance, excellent basic science results in the field of cytokines in psoriasis have been generated for IFN-α, IFN-γ, IL-20, or IL-22, but their *in vivo* neutralization with monoclonal antibodies in clinical trials did not improve psoriasis severity (4–7), indicating that those cytokines, despite their presence in the lesions, are not suitable for patients' treatment.

When studying human chronic cutaneous inflammatory diseases, an alternative approach focused on the human regional cutaneous immune system in disease would allow to focus more closely on the immunological mechanism of disease that takes place within the skin, the proper organ where the pathological process is carried out. The presence of the skin associated lymphoid tissue (SALT) was proposed by J. Wayne Streilein based on the existence of some T cells with special function related only to the skin (8). The Cutaneous Lymphocyte-associated Antigen (CLA) identifies the subset of effector memory T cells with skin tropism that recirculates between skin and blood during cutaneous inflammation and reflects immunological abnormalities present in the skin. The CLA molecule is a carbohydrate modification of the platelet selectin ligand-1 (PSGL-1) (9), which binds both endothelial cell and platelet selectins (E-selectin and P-selectin) molecules expressed on post-capillary venules in the skin. Its expression is induced on CD45R0^+^ memory T cells at skin draining lymph nodes (10) and it is present in more than 90% of cutaneous infiltrating T cells but in <20% of T cells in other peripheral tissues. Interestingly, a proportion of CLA-expressing T cells can be also found in circulation, representing around 15% of human circulating T cells (10). Indeed, the fact that CLA^+^ memory T cells recirculate between skin and blood was evidenced by action of efalizumab (a monoclonal antibody targeting the CD11a subunit of LFA-1 molecule that blocked lymphocyte extravasation toward the skin) in psoriasis (11) and atopic dermatitis (12) patients, which proved clinical improvement but also resulted in a lymphocytosis effect of CLA^+^ T cells that caused relapses after treatment discontinuation (13). The recirculating capacity of CLA^+^ memory T cells is key to their value in translational research, due to their ability to reflect the immune abnormalities found in numerous human cutaneous conditions such as psoriasis, atopic dermatitis, contact dermatitis, drug-induced allergic reactions, vitiligo, herpes simplex, rosacea, cutaneous T cell lymphoma, or alopecia areata (14, 15).

In order to properly study the regional cutaneous immune system in psoriasis, the use of clinical material from non-treated patients provides clear advantages as it is commented below. With this purpose, we have established a new *ex vivo* coculture model made of lymphoid and epidermal cells from the same patient (16) that integrates relevant elements of psoriasis immunopathology: skin-associated memory T cells (CLA^+^ T cells) isolated from blood samples, autologous lesional epidermal cells disaggregated from biopsies from the same patient and a clinically relevant trigger of the disease and flares (such as *Streptococcus pyogenes*). When circulating CLA^+^ T cells and autologous lesional epidermal cells cocultures are activated by *Streptococcus pyogenes* extract (SE), specific Th17/Th1/Th22 responses occurs, together with production of disease relevant chemokines (CXCL9, CXCL10, CXCL11), which is not the case in CLA^−^ T cells cocultures or in unstimulated basal conditions. Interestingly, the levels of IL-17A and other cytokines expressed by CLA^+^ T cells in response to SE are highly correlated with serum anti-Streptolysin O antibody titer (ASO), that is commonly used to assess patients recent infection by *S. pyogenes* in daily clinical practice. The fact that ASO levels and CLA^+^ T cells-dependent IL-17A, IFN-γ, and IL-22 production are directly associated shows how these cytokines can be measured *ex vivo* and integrated with the patient clinical features and environmental exposure to *S. pyogenes*.

In the following subsections, information on cytokines in psoriasis immunopathology is provided from the prism of T cells that are related to the cutaneous immune system. These results have been generated using clinical material (blood and skin biopsies) from non-treated psoriasis patients.

## Influence of Psoriatic Inflammatory Environment on CLA-Dependent IL-17A and IL-17F Production

Memory T cells present in the psoriatic lesion are exposed to the cytokine inflammatory environment and this may influence the effector functions of the IL-17 cytokines, that are clinically relevant mediators in psoriasis. Since peripheral CLA^+^ memory T cells recirculate between skin and blood during psoriasis and reflect the immunological abnormalities present in the skin, they can be used to study the lesional inflammatory environmental cytokines. The presence of IL-15 and IL-23 is increased in lesional skin from psoriasis patients. Psoriatic epidermal keratinocytes show increased expression of both IL-15 and IL-15R (17). Similarly, injured keratinocytes but also dendritic cells have been proved to produce increased amounts of IL-23 in psoriatic lesions (18). Besides, both pro-inflammatory cytokines are linked to Th17 biology. IL-15 is known to induce IL-17 secretion by T cells and to be critical for maintaining memory Th17 cells (19), whilst IL-23 is considered the “master regulator” of Th17 cell differentiation and development. Single nucleotide polymorphisms (SNPs) associated with psoriasis have been also described for both IL-15 and IL-23 (20–22). However, despite their known connection to psoriasis pathogenesis, it was not until recently that the cooperation between these two cytokines has been addressed. Our group has shown that IL-15 and IL-23 act synergistically on cocultures of CLA^+^ memory T cells and autologous epidermal cells to produce significantly increased levels of IL-17F and IL-17A in psoriasis when compared to CLA^−^ T cells cocultures (23). Importantly, this synergy was not observed for lesional resident memory T cells or in cocultures from healthy individuals, pointing out the relevance of circulating CLA^+^ T cells in psoriasis pathogenesis. Altogether, it is a clear example of how the proinflammatory milieu in psoriatic lesions influences specifically skin-tropic memory T cells and induces a Th17 response contributing to the maintenance of the disease.

## CLA^+^ T Cells in Psoriasis Produce More IL-17F Than IL-17A

The IL-17 family comprises six structurally related members (named from A to F) that act as homodimers, except for IL-17A and IL-17F that can form heterodimers together. In psoriasis, IL-17A is pointed out as the most important mediator (24), which is supported by the effectiveness of its blockade in patients. However, its principal homolog, IL-17F, has been also proved to be increased in lesional skin and blood from psoriasis patients (25–27). From our experience, circulating CLA^+^ memory T cells activated with a relevant disease trigger, such as *Streptococcus pyogenes*, and in the presence of lesional epidermal cells, secrete a higher amount of IL-17F than IL-17A *in vitro* (28, 29). Similarly, increased CLA^+^ memory T cell-dependent IL-17F response was observed when stimulating the cocultures with *Candida albicans* (30) or the combination of the pro-inflammatory cytokines IL-15 and IL-23 (23). As such, CLA^+^ memory T cells should be considered an important source of IL-17F in psoriasis. Because IL-17A and IL-17F homodimers and heterodimer share their signaling receptor, constituted by the IL-17RA and IL-17RC subunits, they induce similar gene expression patterns. Nonetheless, the relevance of IL-17F in psoriasis pathogenesis has been shown in patients and animal models. For example, IL-17F knock-out (KO) mice are more resistant to imiquimod-induced psoriasiform inflammation than the IL-17A KO counterparts (31). In humans, the rapid clinical efficacy achieved by brodalumab, a monoclonal antibody targeting the IL-17RA subunit (that is part of the receptor for IL-17A, IL-17F, IL-17A/F heterodimer, and IL-25) first showed the advantages of targeting beyond IL-17A (32, 33). Most importantly, the promising results of the phase 2 and phase 3 clinical trials studying the nanobody sonelokimab and the monoclonal antibody (mAb) bimekizumab, respectively, both of which target IL-17A, IL-17F, and IL-17A/F, further highlight the clinical relevance of IL-17F in psoriasis patients (34, 35).

## IL-9 Is Not Produced Transiently by CLA^+^ T Cells in Human Psoriasis

In humans, IL-9-secreting CD4^+^ T cells, namely Th9, have been described as a distinct T cell population with preferential skin tropism (36). In this work, CLA^+^ Th9 cells were transiently induced by *Candida albicans*-pulsed monocytes particularly but also after non-physiological polyclonal activation, supporting the association of Th9 cells to the skin regardless of their antigen specificity and using lymphocytes from healthy subjects. This transitory Th9 induction peaked just before the increase of IL-17 and IFN-γ induction, suggesting a paracrine regulation by IL-9 that was further confirmed by its own neutralization. More interestingly, a significant increase of IL-9^+^ cells, mostly CD3^+^ CD4^+^ T cells, was found in lesional psoriatic skin compared to atopic dermatitis and healthy cutaneous tissue.

However, when studying the functional role of IL-9 in psoriasis patients (plaque and guttate forms) in our coculture model (29), we found that, after *S. pyogenes* activation, the kinetic of IL-9 production measured in CLA^+^ T cells and epidermal cells coculture supernatants was similar to those of IL-17A and IFN-γ, progressively increasing over time and dependent on MHC class I and class II presentation, which contrasted with the transient IL-9 induction reported before (36). Interestingly, IL-9 partially enhanced SE-induced IL-17A, but not IFN-γ, secretion by CLA^+^ T cells, as well as it promoted the survival of the skin-homing T cell subset in psoriasis.

Using well-defined clinical samples also allows obtaining information on the relationship of cytokines with patient clinical status. CLA^+^ T cells-derived cytokines are associated with clinical features of skin diseases because they represent a subset of memory T cells that are involved in the regional cutaneous immune system. To support the relevance of our findings regarding the role of IL-9 in psoriasis, it was observed that SE-induced CLA^+^ T cells-dependent IL-9 correlated with psoriasis severity (measured as Psoriasis Area Severity Index, PASI) in patients. Moreover, in guttate psoriasis, the peak in IL-9 production was found on patients having the highest ASO levels indicating higher exposure to *S. pyogenes*.

## Guttate Psoriasis: Where Genetic and Environmental Factors Interact Producing Th17 Response By CLA^+^ T Cells

Guttate psoriasis is a form of psoriasis that is strongly associated with the genetic predisposing allele HLA-Cw6 and is commonly triggered by *Streptococcus pyogenes* throat infections (37). This situation has provided a good opportunity to study genetic and environmental factors influence on cytokine production by CLA^+^ T cells in psoriasis (28). The coculture of CLA^+^ T cells activated with *S. pyogenes* clearly demonstrated that non-treated guttate psoriasis patients produced significantly more IL-17A/F cytokines than IFN-γ. However, the most interesting finding was that guttate psoriasis patients carrying the HLA-Cw6 allele and/or whose flare was produced by *S. pyogenes* pharyngitis, in comparison to guttate psoriasis patients that did not fulfill those criteria, significantly produced more IL-17A/F by CLA^+^ T cells. Similar dominant IL-17 response also induced genes that belong to the IL-17 transcriptome in keratinocytes such as *DEFB4, LCN2, IL-8*, but no to the IFN-γ (*CXCL9, CXCL10*, and *CXCL11*). Such influence of genetic and microbe infection on IL-17 response in guttate psoriasis patients has been clarified by analyzing CLA^+^ T cells.

## Plaque Psoriasis: IL-17 Production and Patients Heterogeneity

Chronic plaque psoriasis, or *psoriasis vulgaris*, is the most common form of psoriasis that is generally diagnosed based on the shape and location of the lesions and the persistent duration of the disease. Heterogeneity among plaque psoriasis patients has been suggested (38) but it is still not well-characterized. Nowadays, plaque psoriasis is considered a single entity in most of the clinical studies yet. However, recent results have suggested that the IL-17 cytokines responses by CLA^+^ T cells may be differentially influenced by environmental factors, particularly by exposure to microbes that could favor IL-17 production in chronic plaque psoriasis.

*Streptococcus pyogenes-*induced IL-17 response by CLA^+^ T cells in psoriasis patients is higher when patients present higher titer of immunoglobulin A against *S. pyogenes* extract (39). We reported that anti-SE IgA values in psoriasis patients (both plaque and guttate forms) are higher than in atopic dermatitis and healthy controls. But, most importantly, we found that increased mucosal exposure to *S. pyogenes* was present in chronic plaque psoriasis patients despite negative ASO titer and no clear association of the current flare with *Streptococcal* infection. This finding is of special interest since there may be multiple cases of plaque psoriasis where the implication of *Streptococcus pyogenes* has been discarded, but the bacteria may still be present at the tonsils and contribute to recurrent flares in those patients. In this regard, a recent population-based cohort study has reported how tonsillectomy diminished the risk of developing psoriasis (40). Future studies should confirm how such heterogeneity may influence the natural history of psoriasis disease and/or how different patients respond to specific treatments.

Moreover, chronic plaque psoriasis patients also present increased exposure to *Candida albicans* (CA) (41, 42), that is a potent inducer of IL-17 cytokines in humans (43). Importantly, immune response against this fungus is mainly mediated by CLA^+^ memory T cells, further supporting the pro-inflammatory loop present at cutaneous lesions in psoriasis patients (30). Interestingly, non-treated chronic plaque psoriasis patients, without clinical signs of candidiasis, present increased levels of both IgA and IgG specific for *C. albicans* in plasma. In particular, CLA-dependent IL-17A and IL-17F responses correlated with *Candida-*specific IgA, but not IgG, only in chronic plaque psoriasis. Additionally, a proteomic study of plasma samples from 114 non-treated plaque psoriasis patients revealed that, those with higher anti-*Candida* IgA levels presented increased levels of proteins involved in antimicrobial humoral response, especially proteins showing anti-candida activity [such as eosinophil cationic protein (ECP/RNASE3), Chitinase-3-like protein 1 (CHI3L1) or azurocidin]. These findings point out the implication of *Candida albicans* in chronic plaque psoriasis, a matter that we believe should be further explored in the clinic.

Altogether, these results support the existence of heterogeneity among chronic plaque psoriasis patients that can influence IL-17 production and, thus, the evolution of the course of psoriasis disease.

## Discussion

**Figure 1:**
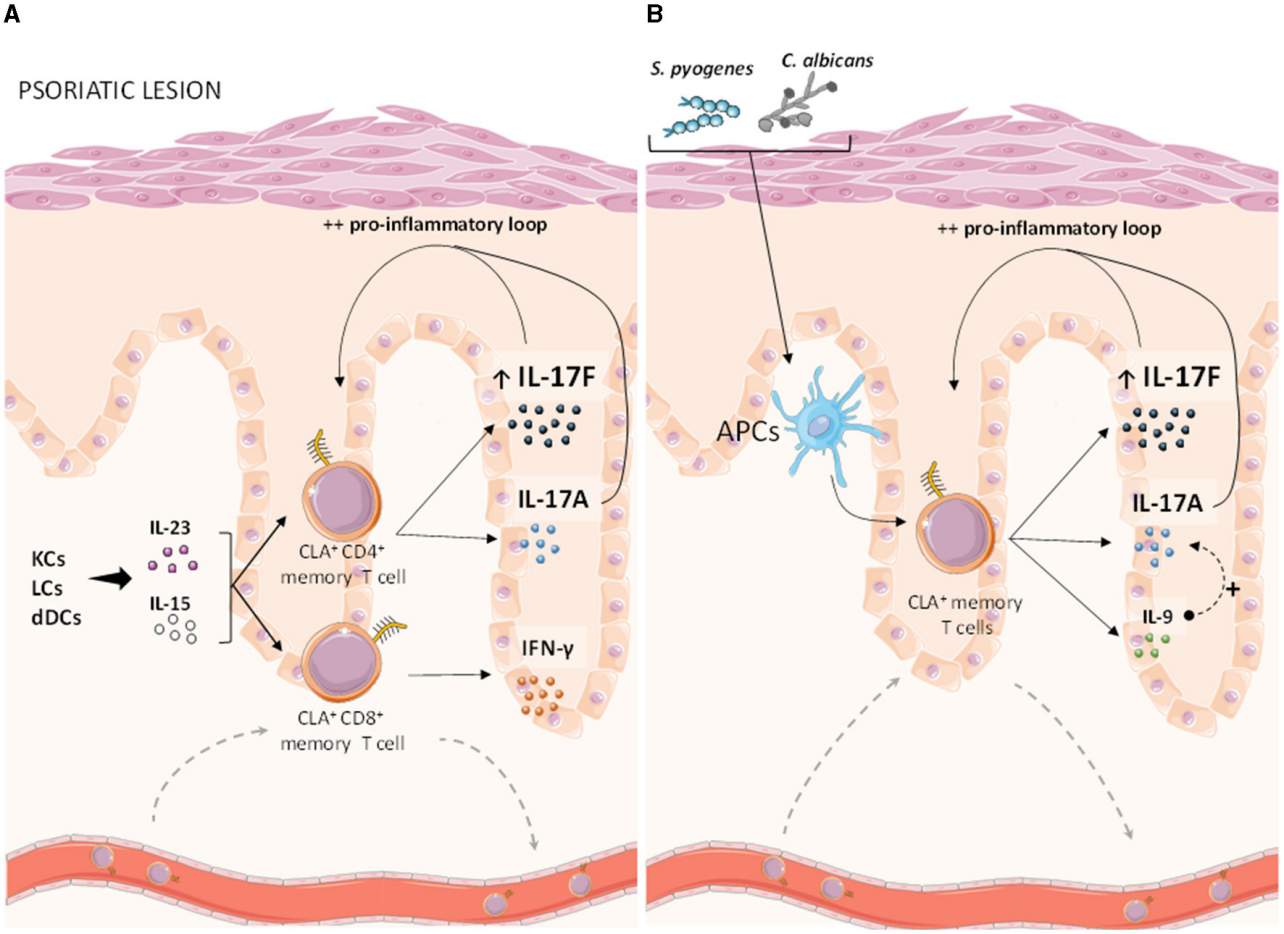
Human CLA^+^ memory T cells and cytokines in psoriasis. As the subset of memory T cells associated to the cutaneous immune system, circulating CLA^+^ T cells are key responders to lesional pro-inflammatory cytokines and relevant microbes that induce important type 17 responses, therefore contributing to the pro-inflammatory loop leading to the appearance of psoriatic lesions. **(A)** IL-15 and IL-23, which are mainly produced by keratinocytes as well as epidermal and dermal dendritic cells, synergistically activate CLA^+^ CD4^+^ memory T cells from psoriasis patients leading to an increased induction of IL-17F and IL-17A cytokines, whereas IFN-γ secretion is likely mediated by CLA^+^ CD8^+^ memory T cells in psoriasis but also in healthy controls. **(B)** *S. pyogenes* and *C. albicans* antigens, presented by epidermal antigen presenting cells, have been proved to induce significantly higher IL-17A/F and IL-9 responses by CLA^+^ memory T cells in psoriasis patients, compared to CLA^−^ T cells. Besides, *S. pyogenes-*induced IL-9 has shown to enhance IL-17A responses, further reinforcing the pro-inflammatory loop underlying psoriasis lesions. CLA, cutaneous leukocyte-associated antigen; KCs, keratinocytes; LCs, Langerhans cells; dDCs, dermal dendritic cells; APCs, antigen presenting cells. This figure was created using Servier Medical Art (smart) images.

In plaque and guttate forms psoriasis, the IL-17 response derived from CLA^+^ T cells is clearly influenced by different factors that are related to patient features and that can be studied *ex vivo* using circulating memory T cells belonging to the regional cutaneous immune system (Figure 1). Using clinical samples from non-treated patients has allowed relating *in vitro* cytokine responses with clinical data in a translational way. For example, these studies reported increased production of IL-17F over IL-17A by skin associated T lymphocytes, which is reflected in the clinic as the greatest response of bimekizumab (neutralizing both IL-17A/F) (34). Also, this approach has shown that guttate psoriasis patients positive for the HLA-C predisposing allele and/or the ASO perform increased cutaneous pro-inflammatory response by CLA^+^ T cells. In the end, how CLA^+^ memory T cells are the link between relevant disease triggers and the increased IL-17 response observed in psoriatic skin.

Mouse models or complex *in vitro* systems, although they are key to study basic pathogenic mechanisms, do not integrate elements that can influence IL-17 responses in patients such as the genetic background or their exposure to microbes able to induce IL-17 responses. How these factors may influence patients response to treatment, the natural history of disease or the existence of psoriasis endotypes, if any, is still an open question in this field. In this regard, the possible role of CLA^+^ memory T cells in the development of psoriatic arthritis (PsA) was early explored but discarded, due to the absence of CLA^+^ memory lymphocytes within synovial tissue (44, 45). The relevance of heterogeneity in chronic plaque psoriasis due to different immunoglobulins specific for microorganisms and CLA^+^ T cells-dependent IL-17A/F responses is particularly interesting since these cytokines, in contrast to others that are also increased in lesions, are validated as safe and clinically effective targets by the use of neutralizing monoclonal antibodies (46).

Nonetheless, this review presents some limitations, as other types of psoriasis (pustular, palmoplantar or inverse), have not been addressed and the study of additional relevant cytokines that are present within psoriatic lesions, such as TNF-α or IL-36, in the context of CLA^+^ memory T cells remains underexplored. Eventually, these limitations evidence how much research is still needed to completely unveil the intricate between cytokines and the cutaneous immune system in the context of psoriasis. Any translational approach that helps to better characterize cytokine biology in the context of the cutaneous immune response will be essential to fully understand patients responses to current therapies and to continue developing novel strategies in the future.

## Author Contributions

CJ-G: writing and figure design. LS, IG-J, MF, and RP: writing. LS-B: IP project founding, writing, and manuscript conceptualization. All authors contributed to the article and approved the submitted version.

## Conflict of Interest

The authors declare that the research was conducted in the absence of any commercial or financial relationships that could be construed as a potential conflict of interest.

## Publisher's Note

All claims expressed in this article are solely those of the authors and do not necessarily represent those of their affiliated organizations, or those of the publisher, the editors and the reviewers. Any product that may be evaluated in this article, or claim that may be made by its manufacturer, is not guaranteed or endorsed by the publisher.
